# Crystal structure and Hirshfeld surface analysis of 3-cyano­phenyl­boronic acid

**DOI:** 10.1107/S2056989018003146

**Published:** 2018-03-02

**Authors:** A. Jaquelin Cárdenas-Valenzuela, Gerardo González-García, Ramón Zárraga- Nuñez, Herbert Höpfl, José J. Campos-Gaxiola, Adriana Cruz-Enríquez

**Affiliations:** a Facultad de Ingeniería Mochis, Universidad Autónoma de Sinaloa, Fuente de Poseidón y Prol. A. Flores S/N, CP 81223, C.U. Los Mochis, Sinaloa, México; b Departamento de Química, División de Ciencias Naturales y Exactas, Campus Guanajuato, Universidad de Guanajuato, Sede Noria Alta, Noria Alta S/N, Col. Noria Alta, CP 36050, Guanajuato, Gto., México; c Centro de Investigaciones Químicas, Instituto de Investigación en Ciencias Básicas y Aplicadas, Universidad Autónoma del Estado de Morelos, Av. Universidad 1001, CP 62209, Cuernavaca, Morelos, México

**Keywords:** crystal structure, boronic acid, hydrogen bonding, offset π–π inter­actions, Hirshfeld surface analysis

## Abstract

In the title compound, C_7_H_6_BNO_2_, the mean plane of the –B(OH)_2_ group is twisted by 21.28 (6)° relative to the cyano­phenyl ring mean plane. In the crystal, mol­ecules are linked by O—H⋯O and O—H⋯N hydrogen bonds, forming chains propagating along the [101] direction. Offset π–π and B⋯π stacking inter­actions link the chains, forming a three-dimensional network. Hirshfeld surface analysis shows that van der Waals inter­actions constitute a further major contribution to the inter­molecular inter­actions, with H⋯H contacts accounting for 25.8% of the surface.

## Abstract 2

In the title boronic acid derivative, the mean plane of the –B(OH)_2_ group is twisted by 21.28 (6)° relative to the cyano­phenyl ring mean plane. In the crystal, mol­ecules are linked by O—H⋯O and O—H⋯N hydrogen bonds, forming chains propagating along [101].

## Chemical context   

Boron-containing compounds and particularly aryl­boronic acid are an important class of compounds in the fields of organic and medicinal chemistry, and have played a role in the development of modern organic synthesis, macromolecular chemistry, crystal engineering and mol­ecular recognition (Fujita *et al.*, 2008 ▸; Severin, 2009 ▸). As a result of their peculiar dynamic covalent reactivity with alcohols (Jin *et al.*, 2013 ▸), aryl­boronic acids and their dehydrated derivatives enable the self-assembly of a large variety of architectures resulting from boronate esterification (Takahagi *et al.* 2009 ▸) as well as boroxine (Côté *et al.*, 2005 ▸) and spiro­borate formation (Du *et al.*, 2016 ▸).

**Figure 1:**
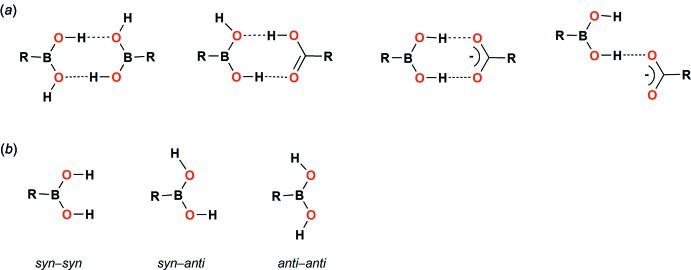
(*a*) Neutral and charge-assisted homo- and heterodimeric hydrogen-bonding motifs involving boronic acids. (*b*) Conformations of the boronic acid moiety.

Boronic acids form neutral and charge-assisted homo- and heterodimeric hydrogen-bonding patterns resembling characteristics similar to those found for carb­oxy­lic acids (see Fig. 1 ▸ *a*). However, the –B(OH)_2_ moiety contains two O—H hydrogen-bond donors and can, thus, form two O—H⋯*X* hydrogen bonds and adopt different conformations (see Fig. 1 ▸ *b*). This enables the generation of hydrogen-bonding networks with increased dimensionality (one to three dimensions) in the solid state (Fournier *et al.*, 2003 ▸; Madura *et al.*, 2015 ▸; Georgiou *et al.*, 2017 ▸). In recent years, boronic acids have also been explored in the context of forming multicomponent mol­ecular complexes with organic carb­oxy­lic acids (–COOH), amides (–CONH_2_), alcohols (–OH) and pyridines, which are based on mol­ecular recognition processes (Rodríguez-Cuamatzi *et al.*, 2005 ▸; Madura *et al.*, 2014 ▸; Hernández-Paredes *et al.*, 2015 ▸; Campos-Gaxiola *et al.*, 2017 ▸; Pedireddi & Lekshmi, 2004 ▸; Vega *et al.*, 2010 ▸; TalwelkarShimpi *et al.*, 2016 ▸). As part of our ongoing studies in this area, we report herein on the mol­ecular and crystal structures of 3-cyano­phenyl­boronic acid, I[Chem scheme1]. In addition, a Hirshfeld surface analysis was performed to visualize and qu­antify the inter­molecular inter­actions in the crystal structure of compound (I).
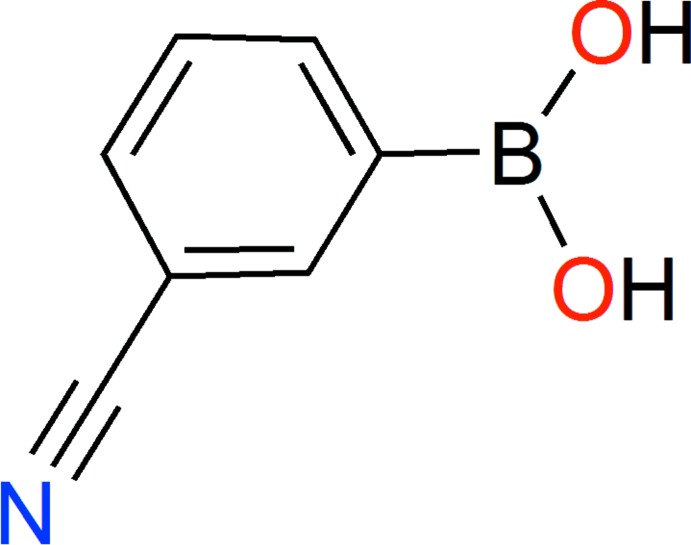


## Structural commentary   

**Figure 2:**
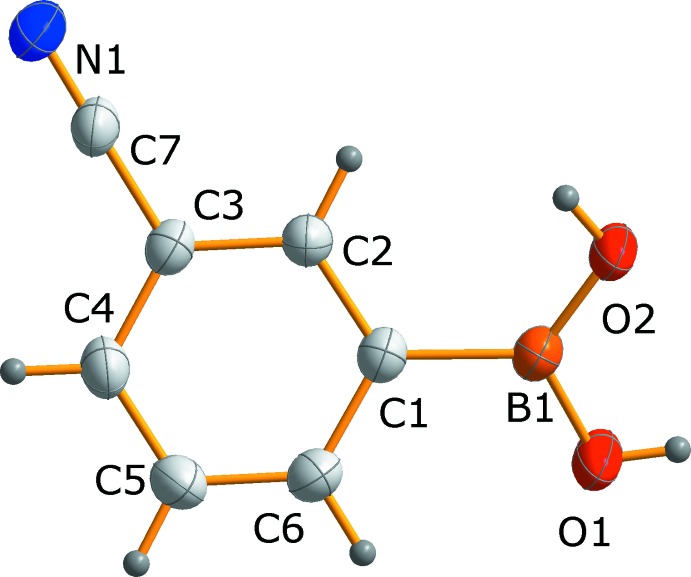
The mol­ecular structure of the title compound (I), with the atom labeling. Displacement ellipsoids are drawn at the 50% probability level.

The mol­ecular structure of the title compound (I) is illustrated in Fig. 2 ▸. It can be seen that the –B(OH)_2_ group adopts the most preferred *syn–anti* conformation (Lekshmi & Pedireddi, 2007 ▸). As a result of the H⋯H repulsion between the *endo*-oriented B—OH hydrogen and the C—H hydrogen in position 2 of the aromatic ring, the –B(OH)_2_ mean plane is twisted by 21.28 (6)° relative to the cyano­phenyl ring mean plane. This torsion disables intra­molecular C—H⋯O hydrogen bonding between the oxygen atom of the *exo*-oriented B—OH function and weakens the B—C π–π bonding inter­actions (Durka *et al.*, 2012 ▸). The B1—O1, B1—O2 and B1—C1 bond lengths are 1.3455 (17), 1.3661 (18) and 1.5747 (18) Å, respectively. For comparison, in coplanar triphenyl boroxine the B—C bond lengths range from 1.544 (4) to 1.549 (4) Å (Brock *et al.*, 1987 ▸). The C≡N bond length of 1.1416 (18) Å is typical for a bond with triple-bond character.

## Supra­molecular features   

**Figure 3:**
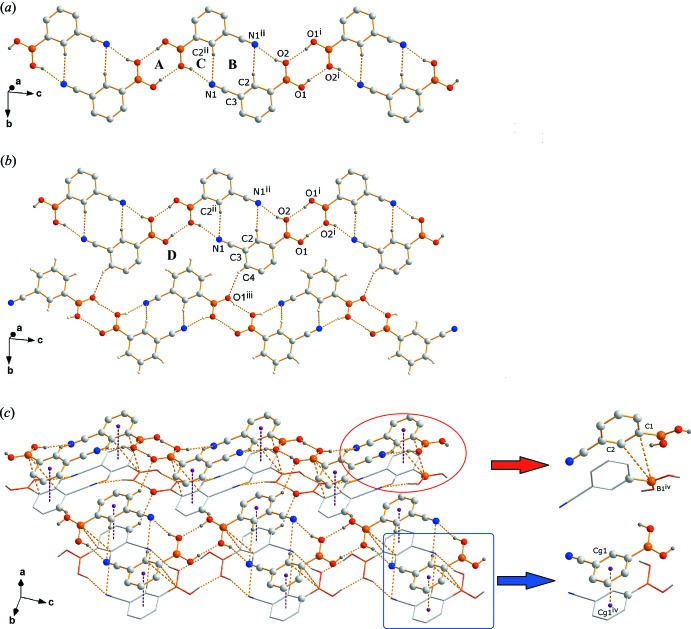
Hydrogen-bonding motifs and π–π inter­actions found in the crystal structure of (I). [Symmetry codes: (i) 2 − *x*, 1 − *y*, 1 − *z*; (ii) 1 − *x*, 1 − *y*, −*z*; (iii) −1 + *x*, 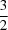 − *y*, −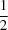 + *z*; (iv) −1 + *x*, *y*, *z*.]

In the crystal of (I), the boronic acid mol­ecules are in the first instance associated to form chains through two well-known double-bridged homodimeric motifs based on a –BOH⋯O(H)B– [motif **A**; graph set 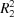
(8)] and C—H⋯N≡C hydrogen bonds [motif **B**; graph set 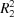
(10)]. This hydrogen-bonding pattern is strengthened further by a –BOH⋯N≡C contact [motif **C**; graph set 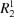
(7)] (Fig. 3 ▸ *a*, Table 1 ▸). In comparison to the crystal structure of 4-cyano­phenyl­boronic acid, where the chains are almost linear (TalwelkarShimpi *et al.*, 2017 ▸), in (I) they have a pronounced zigzag topology. The O1⋯O2^i^, C2⋯N1^ii^ and O2⋯N1^ii^ separations in motifs **A**, **B** and **C** are 2.796 (1), 3.452 (2) and 2.909 (2) Å, respectively (Table 1 ▸), and are similar to distances reported for related systems (Rodríguez-Cuamatzi *et al.*, 2005 ▸; TalwelkarShimpi *et al.*, 2017 ▸). Within the crystal structure, neighboring tapes are linked through additional C—H⋯O contacts to give an overall two-dimensional network running parallel to (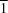
01) with macrocyclic motifs **D** [graph set 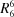
(26)], see Fig. 3 ▸ *b*. The C4⋯O1^iii^ distance is 3.469 (2) Å, see Table 1 ▸. The resulting 2D networks stack in a parallel fashion to form a layered 3D structure based on offset π–π inter­actions between adjacent 3-cyano­phenyl­boronic acid mol­ecules [*Cg*⋯*Cg* ^iv^ = 3.8064 (8) Å; slippage 1.38 Å; symmetry code (iv) = −1 + *x*, *y*, *z*] and η^2^-type B⋯π contacts with B⋯C distances of 3.595 (2) and 3.673 (2) Å (Fig. 3 ▸ *c*). Similar inter­actions are also depicted in mol­ecular crystals formed between 1,4-benzene­diboronic acid and aromatic amine *N*-oxides (Sarma & Baruah, 2009 ▸; Sarma *et al.*, 2011 ▸).

## Hirshfeld surface analysis   

**Figure 4:**
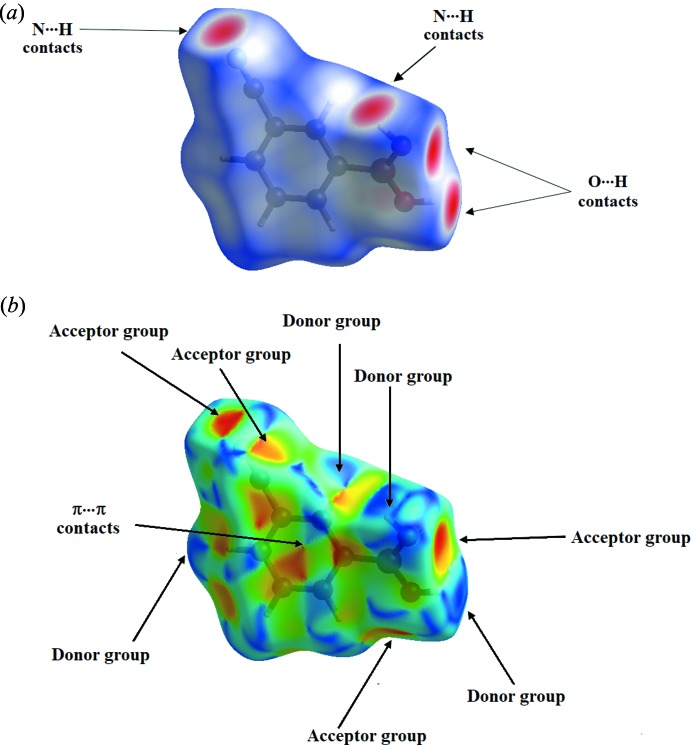
Hirshfeld surfaces for compound (I), mapped with *d*_norm_ (top) and shape-index (bottom).

Hirshfeld surfaces and fingerprint plots were generated for (I) based on the crystallographic information file (CIF) using *CrystalExplorer* (Hirshfeld, 1977 ▸; McKinnon *et al.*, 2004 ▸). Hirshfeld surfaces enable the visualization of inter­molecular inter­actions by different colors and color intensity, representing short or long contacts and indicating the relative strength of the inter­actions. Fig. 4 ▸ shows the Hirshfeld surface of the title compound mapped over *d*_norm_ (−0.60 to 0.90 Å) and the shape-index (−1.0 to 1.0 Å). In the *d*_norm_ map, the vivid red spots in the Hirshfeld surface are due to short normalized O⋯H and N⋯H distances corresponding to O—H⋯O and O—H⋯N inter­actions. The white spots represent the contacts resulting from C—H⋯N hydrogen bonding (Fig. 4 ▸ *a*). On the shape-index surface for compound (I), convex blue regions represent hydrogen-donor groups and concave red regions represent hydrogen-acceptor groups. The –B(OH)_2_ group behaves simultaneously as a donor and an acceptor, meanwhile the –C≡N group is an acceptor only. The occurrence of offset π–π inter­actions is indicated by adjacent red and blue triangles (Fig. 4 ▸ *b*).

**Figure 5:**
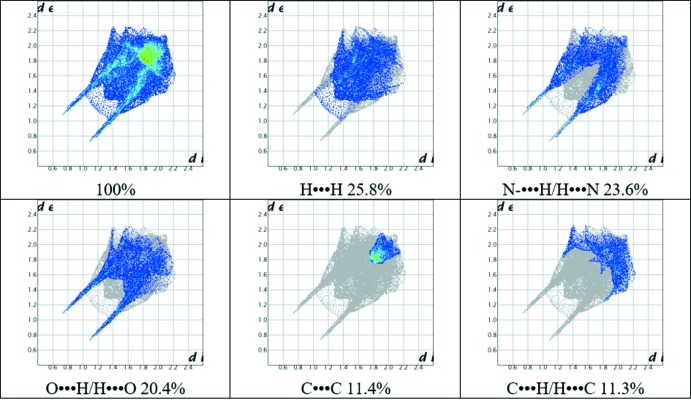
Two-dimensional fingerprints of compound (I), showing H⋯H, N⋯H, O⋯H, C⋯C and C⋯H close contacts.

The two-dimensional fingerprint plots qu­antify the contributions of each type of non-covalent inter­action to the Hirshfeld surface (McKinnon *et al.*, 2007 ▸). The major contribution with 25.8% of the surface is due to H⋯H contacts, which represent van der Waals inter­actions, followed by N⋯H and O⋯H inter­actions, which contribute 23.6 and 20.4%, respectively (these contributions are observed as two sharp peaks in the plot of Fig. 5 ▸). This behavior is usual for strong hydrogen bonds (Spackman & McKinnon, 2002 ▸). Finally, the presence of C⋯C (11.4%) and B⋯C (2.3%) contacts corresponds to the π–π and B⋯π inter­actions, respectively, established in the crystal structure analysis section.

## Experimental   

3-Cyano­phenyl­boronic acid and the solvent used in this work are commercially available and were used without further purification. For single-crystal growth, a solution of 3-cyano­phenyl­boronic acid (0.050 g) in 5 ml of ethanol was heated to reflux for 15 min. The solution was left to evaporate slowly at room temperature, giving after one week colorless crystals suitable for single-crystal X-ray diffraction analysis.

## Refinement   

Crystal data, data collection and structure refinement details are summarized in Table 2 ▸. Hydrogen atoms were positioned geometrically (O—H = 0.82 Å and C—H = 0.93 Å) and refined using a riding model, with *U*_iso_(H) = 1.2*U*_eq_(C) and 1.5*U*_eq_(O).

## Supplementary Material

Crystal structure: contains datablock(s) Global, I. DOI: 10.1107/S2056989018003146/su5425sup1.cif

Structure factors: contains datablock(s) I. DOI: 10.1107/S2056989018003146/su5425Isup2.hkl

Supporting information file. DOI: 10.1107/S2056989018003146/su5425Isup3.cml

CCDC reference: 1825335

Additional supporting information:  crystallographic information; 3D view; checkCIF report

## Figures and Tables

**Table 1:** Hydrogen-bond geometry (Å, °)

*D*—H⋯*A*	*D*—H	H⋯*A*	*D*⋯*A*	*D*—H⋯*A*
O1—H1⋯O2^i^	0.82	1.98	2.796 (1)	170
O2—H2⋯N1^ii^	0.82	2.12	2.909 (2)	160
C2—H2*A*⋯N1^ii^	0.93	2.71	3.452 (2)	138
C4—H4⋯O1^iii^	0.93	2.67	3.469 (2)	144

Symmetry codes: (i) 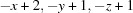; (ii) 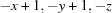; (iii) 
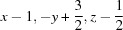
.

**Table 2:** Experimental details

Crystal data	
Chemical formula	C_7_H_6_BNO_2_
*M*_r_	146.94
Crystal system, space group	Monoclinic, *P*2_1_/*c*
Temperature (K)	293
*a*, *b*, *c* (Å)	3.8064 (2), 16.156 (1), 11.4585 (4)
β (°)	93.472 (4)
*V* (Å^3^)	703.36 (6)
*Z*	4
Radiation type	Mo *K*α
μ (mm^−1^)	0.10
Crystal size (mm)	0.48 × 0.25 × 0.20

Data collection	
Diffractometer	Rigaku OD SuperNova Single source at offset EosS2
Absorption correction	Gaussian (*CrysAlis PRO*; Rigaku OD, 2015 ▸)
*T*_min_, *T*_max_	0.992, 0.996
No. of measured, independent and observed [*I* > 2σ(*I*)] reflections	7332, 1434, 1347
*R*_int_	0.023
(sin θ/λ)_max_ (Å^−1^)	0.625

Refinement	
*R*[*F* ^2^ > 2σ(*F* ^2^)], *wR*(*F* ^2^), *S*	0.042, 0.110, 1.09
No. of reflections	1434
No. of parameters	102
H-atom treatment	H-atom parameters constrained
Δρ_max_, Δρ_min_ (e Å^−3^)	0.20, −0.24

Computer programs: *CrysAlis PRO* (Rigaku OD, 2015 ▸), *SUPERFLIP* (Palatinus & Chapuis, 2007 ▸; Palatinus & van der Lee, 2008 ▸; Palatinus *et al.*, 2012 ▸), *SHELXL2016* (Sheldrick, 2015 ▸) and *OLEX2* (Dolomanov *et al.*, 2009 ▸).

## supplementary crystallographic information

### Crystal data

**Table:**

C_7_H_6_BNO_2_	*F*(000) = 304
*M**_r_* = 146.94	*D*_x_ = 1.388 Mg m^−^^3^
Monoclinic, *P*2_1_/*c*	Mo *K*α radiation, λ = 0.71073 Å
*a* = 3.8064 (2) Å	Cell parameters from 4312 reflections
*b* = 16.156 (1) Å	θ = 4.4–29.1°
*c* = 11.4585 (4) Å	µ = 0.10 mm^−^^1^
β = 93.472 (4)°	*T* = 293 K
*V* = 703.36 (6) Å^3^	Block, colourless
*Z* = 4	0.48 × 0.25 × 0.20 mm

### Data collection

**Table:**

Rigaku OD SuperNova Single source at offset EosS2 diffractometer	1434 independent reflections
Radiation source: micro-focus sealed X-ray tube, SuperNova (Mo) X-ray Source	1347 reflections with *I* > 2σ(*I*)
Mirror monochromator	*R*_int_ = 0.023
Detector resolution: 8.0945 pixels mm^-1^	θ_max_ = 26.4°, θ_min_ = 3.6°
ω scans	*h* = −4→4
Absorption correction: gaussian (CrysAlis PRO; Rigaku OD, 2015)	*k* = −20→20
*T*_min_ = 0.992, *T*_max_ = 0.996	*l* = −14→14
7332 measured reflections	

### Refinement

**Table:**

Refinement on *F*^2^	Primary atom site location: iterative
Least-squares matrix: full	Secondary atom site location: difference Fourier map
*R*[*F*^2^ > 2σ(*F*^2^)] = 0.042	Hydrogen site location: inferred from neighbouring sites
*wR*(*F*^2^) = 0.110	H-atom parameters constrained
*S* = 1.09	*w* = 1/[σ^2^(*F*_o_^2^) + (0.0566*P*)^2^ + 0.1839*P*] where *P* = (*F*_o_^2^ + 2*F*_c_^2^)/3
1434 reflections	(Δ/σ)_max_ < 0.001
102 parameters	Δρ_max_ = 0.20 e Å^−^^3^
0 restraints	Δρ_min_ = −0.24 e Å^−^^3^

### Special details

**Table:**

Geometry. All esds (except the esd in the dihedral angle between two l.s. planes) are estimated using the full covariance matrix. The cell esds are taken into account individually in the estimation of esds in distances, angles and torsion angles; correlations between esds in cell parameters are only used when they are defined by crystal symmetry. An approximate (isotropic) treatment of cell esds is used for estimating esds involving l.s. planes.

### Fractional atomic coordinates and isotropic or equivalent isotropic displacement parameters (Å^2^)

**Table:**

	*x*	*y*	*z*	*U*_iso_*/*U*_eq_	
O2	0.8906 (3)	0.49351 (6)	0.34935 (8)	0.0436 (3)	
H2	0.881271	0.482459	0.279381	0.065*	
O1	0.8143 (3)	0.60701 (6)	0.47187 (8)	0.0507 (3)	
H1	0.900315	0.572989	0.518407	0.076*	
N1	0.0762 (4)	0.58588 (8)	−0.12243 (11)	0.0485 (4)	
C3	0.3424 (3)	0.65006 (8)	0.06942 (10)	0.0307 (3)	
C7	0.1909 (4)	0.61462 (8)	−0.03743 (11)	0.0355 (3)	
C1	0.6246 (3)	0.62961 (8)	0.26214 (10)	0.0302 (3)	
C2	0.4765 (3)	0.59744 (8)	0.15775 (11)	0.0309 (3)	
H2A	0.467006	0.540428	0.146805	0.037*	
C4	0.3561 (4)	0.73542 (8)	0.08286 (12)	0.0367 (3)	
H4	0.267843	0.770116	0.023320	0.044*	
C6	0.6325 (4)	0.71575 (8)	0.27423 (11)	0.0352 (3)	
H6	0.727616	0.738593	0.343630	0.042*	
C5	0.5031 (4)	0.76802 (8)	0.18615 (12)	0.0400 (3)	
H5	0.515094	0.825073	0.196425	0.048*	
B1	0.7827 (4)	0.57324 (9)	0.36442 (12)	0.0336 (3)	

### Atomic displacement parameters (Å^2^)

**Table:**

	*U*^11^	*U*^22^	*U*^33^	*U*^12^	*U*^13^	*U*^23^
O2	0.0679 (7)	0.0371 (5)	0.0242 (5)	0.0103 (5)	−0.0092 (5)	−0.0004 (4)
O1	0.0810 (8)	0.0414 (6)	0.0278 (5)	0.0165 (5)	−0.0120 (5)	−0.0029 (4)
N1	0.0625 (9)	0.0474 (7)	0.0339 (7)	0.0077 (6)	−0.0115 (6)	−0.0044 (5)
C3	0.0303 (6)	0.0361 (7)	0.0254 (6)	0.0025 (5)	−0.0001 (5)	0.0001 (5)
C7	0.0398 (7)	0.0365 (7)	0.0295 (7)	0.0077 (5)	−0.0031 (5)	0.0028 (5)
C1	0.0304 (6)	0.0332 (7)	0.0267 (6)	0.0014 (5)	−0.0002 (5)	0.0017 (5)
C2	0.0343 (7)	0.0291 (6)	0.0290 (6)	0.0019 (5)	−0.0011 (5)	0.0005 (5)
C4	0.0426 (8)	0.0353 (7)	0.0320 (7)	0.0063 (5)	0.0004 (5)	0.0067 (5)
C6	0.0397 (7)	0.0358 (7)	0.0296 (6)	−0.0019 (5)	−0.0016 (5)	−0.0031 (5)
C5	0.0520 (8)	0.0287 (7)	0.0390 (8)	0.0007 (6)	0.0009 (6)	0.0001 (5)
B1	0.0385 (8)	0.0347 (8)	0.0268 (7)	0.0006 (6)	−0.0038 (6)	0.0010 (5)

### Geometric parameters (Å, º)

**Table:**

O2—B1	1.3661 (18)	C1—C2	1.3917 (17)
O1—B1	1.3455 (17)	C1—C6	1.3987 (18)
N1—C7	1.1416 (18)	C1—B1	1.5747 (18)
C3—C7	1.4401 (18)	C4—C5	1.3825 (19)
C3—C2	1.3948 (17)	C6—C5	1.3834 (19)
C3—C4	1.3882 (19)		
			
C2—C3—C7	119.00 (12)	C1—C2—C3	120.50 (12)
C4—C3—C7	119.98 (11)	C5—C4—C3	118.95 (12)
C4—C3—C2	121.02 (12)	C5—C6—C1	122.04 (12)
N1—C7—C3	178.81 (15)	C4—C5—C6	119.98 (12)
C2—C1—C6	117.51 (11)	O2—B1—C1	123.75 (11)
C2—C1—B1	122.70 (11)	O1—B1—O2	119.15 (12)
C6—C1—B1	119.79 (11)	O1—B1—C1	117.10 (12)
			
C3—C4—C5—C6	−0.2 (2)	C2—C1—B1—O1	−159.37 (13)
C7—C3—C2—C1	−179.96 (12)	C4—C3—C2—C1	0.64 (19)
C7—C3—C4—C5	−179.92 (12)	C6—C1—C2—C3	0.00 (18)
C1—C6—C5—C4	0.9 (2)	C6—C1—B1—O2	−158.17 (13)
C2—C3—C4—C5	−0.5 (2)	C6—C1—B1—O1	21.14 (19)
C2—C1—C6—C5	−0.8 (2)	B1—C1—C2—C3	−179.50 (12)
C2—C1—B1—O2	21.3 (2)	B1—C1—C6—C5	178.76 (13)

### Hydrogen-bond geometry (Å, º)

**Table:**

*D*—H···*A*	*D*—H	H···*A*	*D*···*A*	*D*—H···*A*
O1—H1···O2^i^	0.82	1.98	2.796 (1)	170
O2—H2···N1^ii^	0.82	2.12	2.909 (2)	160
C2—H2*A*···N1^ii^	0.93	2.71	3.452 (2)	138
C4—H4···O1^iii^	0.93	2.67	3.469 (2)	144

Symmetry codes: (i) −*x*+2, −*y*+1, −*z*+1; (ii) −*x*+1, −*y*+1, −*z*; (iii) *x*−1, −*y*+3/2, *z*−1/2.
